# Genome Sequence of *Burkholderia cenocepacia* H111, a Cystic Fibrosis Airway Isolate

**DOI:** 10.1128/genomeA.00298-14

**Published:** 2014-04-10

**Authors:** Aurelien Carlier, Kirsty Agnoli, Gabriella Pessi, Angela Suppiger, Christian Jenul, Nadine Schmid, Burkhard Tümmler, Marta Pinto-Carbo, Leo Eberl

**Affiliations:** a Department of Microbiology, Institute of Plant Biology, University of Zürich, Zürich, Switzerland; b Department of Pediatric Pneumology, Hannover Medical School, Hannover, Germany

## Abstract

The *Burkholderia cepacia* complex (BCC) is a group of related bacterial species that are commonly isolated from environmental samples. Members of the BCC can cause respiratory infections in cystic fibrosis patients and immunocompromised individuals. We report here the genome sequence of *Burkholderia cenocepacia* H111, a well-studied model strain of the BCC.

## GENOME ANNOUNCEMENT

The *Burkholderia cepacia* complex (BCC) is a group of 18 closely related bacterial species, which can cause life-threatening disease in immunocompromised and cystic fibrosis (CF) patients (1, 2). BCC species are commonly found in soil or associated with plants (3–5). *Burkholderia cenocepacia* H111 is a member of the BCC that was isolated from a sputum sample from a CF patient (6). While closely related, *B. cenocepacia* H111 does not belong to the epidemic ET12 lineage, from which three genomes (strains J2315, BC7, and K56-2) have been sequenced (7, 8). Strains of the ET12 lineage are highly transmissible and have resulted in high mortality among infected CF patients (9, 10). In contrast, the CF patient infected with *B. cenocepacia* H111 did not show acute symptoms. The infection cleared after a 6-month coinfection period with *Pseudomonas aeruginosa* (6). *B. cenocepacia* H111 is sensitive to several antibiotics, is easily amenable to genetic manipulation, and its physiology and virulence in various infection models are well studied (11–16). Together, these properties make its genome a valuable addition to the growing number of sequenced BCC genomes.

Genomic DNA was prepared according to Wilson (17). A combination of Roche 454 GS-FLX Titanium 3-kb-span paired-end libraries (~25× coverage), Roche 454 GS-GLX Titanium fragment reads (~25× coverage), and Illumina 36-bp fragments (50× coverage) were assembled into 71 contigs using the CLC Genomics Workbench version 5.0 software (CLC bio, Aarhus, Denmark). To fill the small gaps in the assembly that were probably caused by ambiguities in G+C-rich regions of the genome, we sequenced a large-insert library (8 to 12 kb) using the PacBio RS II kit to approximately 30× coverage (Pacific Biosciences, Menlo Park, CA, USA). We used the AHA algorithm from the SMRT Portal (Pacific Biosciences) to close all the gaps and generate high-quality circular consensus sequences of the 2 chromosomes and the megaplasmid pC3 that comprise the *B. cenocepacia* H111 genome.

Chromosomes 1 and 2 in H111 have sizes of 3.57 Mb and 3.10 Mb, respectively, and are slightly smaller than those of the closely related *B. cenocepacia* J2315 strain (3.83 Mb and 3.19 Mb, respectively) (8). Several large genomic islands are missing from chromosome 1 of H111 (e.g., BcenGI5, BcenGI6, and BenGI8 with sizes of 92 kb, 34 kb, and 121 kb, respectively), accounting for the smaller size. A 102-kb region that contains the *B. cenocepacia* pathogenicity island *cci* (18), the type IV secretion system genomic island (8), and the low-oxygen-activated locus *lxa* (19) is absent from chromosome 2 of *B. cenocepacia* H111. Conversely, the facultative pC3 replicon is larger than that of *B. cenocepacia* J2315 at 1.04 Mb versus 0.88 Mb. The pC3 replicon codes for specific traits, such as virulence and stress resistance, and is less conserved among BCC members than chromosomes 1 and 2 (20, 21).

The genome was annotated using the RAST pipeline (22). The results of the automated annotation were augmented and curated using the Artemis software package (23). The genome contains 6,933 open reading frames, which is comparable to the gene content of other *B. cenocepacia* genomes.

### Nucleotide sequence accession numbers.

The sequences of the three replicons of *B. cenocepacia* H111 have been deposited at DDBJ/EMBL/GenBank under the accession no. HG938370, HG938371, and HG938372.
